# 2,2′,4,4′,6,6′-Hexamethyl-*N*-(3-phthalimidoprop­yl)-*N*,*N*′-(propane-1,3-di­yl)dibenzene­sulfonamide

**DOI:** 10.1107/S1600536808036003

**Published:** 2008-11-08

**Authors:** Yu-Xia Wang, Peng-Fei Cheng, Chao-Jie Wang

**Affiliations:** a College of Chemistry and Chemical Engineering, Henan University, Kaifeng 475004, People’s Republic of China

## Abstract

In the title compound, C_32_H_38_N_3_O_6_S_2_, an inter­mediate in the synthesis of polyamine drugs, the dihedral angle between the phenyl rings of the two 2,4,6-trimethyl­benzene­sulfonyl groups is 27.1 (3)°. In the crystal structure, mol­ecules are linked by inter­molecular N—H⋯O hydrogen bonds, thereby forming an infinite one-dimensional chain propagating along [010].

## Related literature

Polyamines are essential growth factors for cells, existing mainly as polycations at physiological pH, see: Cullis *et al.* (1999 ▶); Seiler *et al.* (1996 ▶); Tsen *et al.* (2008 ▶).
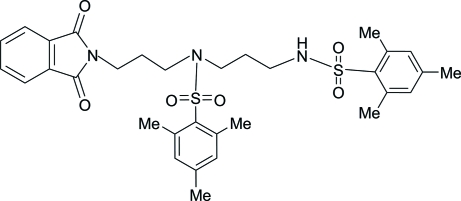


## Experimental

### 

#### Crystal data

C_32_H_39_N_3_O_6_S_2_
*M* *_r_* = 625.78
Monoclinic, 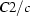

*a* = 32.042 (3) Å
*b* = 9.9782 (8) Å
*c* = 25.105 (2) Å
β = 127.917 (1)°
*V* = 6332.1 (9) Å^3^
*Z* = 8
Mo *K*α radiation
μ = 0.22 mm^−1^
*T* = 296 (2) K
0.18 × 0.15 × 0.13 mm

#### Data collection

Bruker SMART CCD diffractometer
Absorption correction: multi-scan (*SADABS*; Sheldrick, 2001 ▶) *T*_min_ = 0.962, *T*_max_ = 0.973
17128 measured reflections
6212 independent reflections
4574 reflections with *I* > 2σ(*I*)
*R*_int_ = 0.022

#### Refinement

*R*[*F* ^2^ > 2σ(*F* ^2^)] = 0.069
*wR*(*F* ^2^) = 0.219
*S* = 1.09
6212 reflections
388 parameters
44 restraints
H-atom parameters constrained
Δρ_max_ = 0.80 e Å^−3^
Δρ_min_ = −0.57 e Å^−3^

### 

Data collection: *SMART* (Bruker, 2001 ▶); cell refinement: *SAINT-Plus* (Bruker, 2001 ▶); data reduction: *SAINT-Plus*; program(s) used to solve structure: *SHELXS97* (Sheldrick, 2008 ▶); program(s) used to refine structure: *SHELXL97* (Sheldrick, 2008 ▶); molecular graphics: *PLATON* (Spek, 2003 ▶); software used to prepare material for publication: *PLATON*.

## Supplementary Material

Crystal structure: contains datablocks global, I. DOI: 10.1107/S1600536808036003/hb2835sup1.cif

Structure factors: contains datablocks I. DOI: 10.1107/S1600536808036003/hb2835Isup2.hkl

Additional supplementary materials:  crystallographic information; 3D view; checkCIF report

## Figures and Tables

**Table 1:** Hydrogen-bond geometry (Å, °)

*D*—H⋯*A*	*D*—H	H⋯*A*	*D*⋯*A*	*D*—H⋯*A*
N3—H3*B*⋯O5^i^	0.86	2.53	3.192 (4)	134

Symmetry code: (i) 
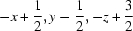
.

**Fig. 1.**
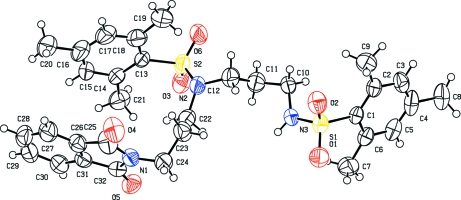
The molecular structure of (I). Displacement ellipsoids for the non-hydrogen atoms are drawn at the 50% probability level.

**Fig. 2.**
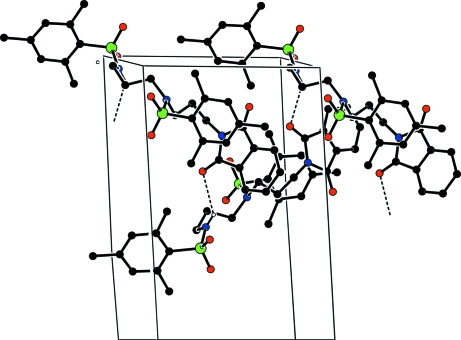
One-dimensional structure of (I), Hydrogen bonds are shown as dashed lines. For clarity, H atoms not involved in hydrogen bonds are omitted.

## supplementary crystallographic information

### Comment

Polyamines are essential growth factors for cells, which exist mainly as polycations at physiological pH (Cullis *et al.*,  1999; Seiler *et al.*,  1996; Tsen *et al.*,  2008). As part of our studies in this area, herein we report the synthesis and structure of the title compound, (I).

The compound (I) consists of a polyamine chain with two 2,4,6-trimethylbenzenesulfonyl group acting as protecting groups (Fig. 1). In the structure of (I), the two phenyl ring of two 2,4,6-trimethylbenzenesulfonyl group are nonparallel due to steric hindrance, charactrtized by a dihedral angel of 27.1 (3) °.

In the crystal, molecules are linked through intermolecular N–H···O hydrogen bonds to construct an infinite one-dimensional chain (Fig. 2 and Table 1).

### Experimental

Propane-1,3-diamine 1.85 g (25 mmol) was dissolved in 2 *M* sodium hydroxide and the solution was cooled to 0 °C, a solution of 2,4,6-trimethylbenzenesulfonyl chloride 10.9 g (50 mmol) in CH_2_Cl_2_ (25 ml) was added dropwise. The reaction mixture was then stirred at room temperture for 18 h. The organic phase was separated from the aqueous phase and washed with 0.5 *M* HCl solution and brine. The CH_2_Cl_2_ layer was dried over sodium sulfate, filtered and the solvent removed *in vacuo*, and the residue purified by chromatography.

A mixture of *N*^1^,*N*^3^—Bis(mesitylenesulfonyl)-1,3-propyl-diamine 1.05 g (2.33 mmol) and 60% NaH (5.35 mmol, 0.22 g) in DMF 20 ml was stirred at 0 °C for 0.5 h, then warmed to room temperature for 0.5 h. *N*-(3-bromopropyl)phthalimide 1.57 g (5.82 mmol) was added and the reaction mixture was stirred at 40 °C for 4 h, then EtoH (2.5 ml) and water (5 ml) were added, the solvent was removed *in vacuo* at 80 °C, the residue was dissolved in CHCl_3_ and washed with water, the organic layer was dried over anhydrous sodium sulfate and filtered, then concentrated *in vacuo*, the residue was purified by chromatography. Colorless rod crystal of (I) were obtained.

### Refinement

The H atoms were positioned geogmetrically (N—H = 0.86 Å, C—H = 0.93–0.97Å) and refined as riding with *U*_iso_(*H*)=1.2*U*_eq_(carrier) or *U*_iso_(*H*)=1.5*U*_eq_(methyl-C).

### Crystal data

**Table:**

C_32_H_39_N_3_O_6_S_2_	*F*_000_ = 2656
*M**_r_* = 625.78	*D*_x_ = 1.313 Mg m^−^^3^
Monoclinic, *C*2/*c*	Mo *K*α radiation λ = 0.71073 Å
Hall symbol: -C 2yc	Cell parameters from 6528 reflections
*a* = 32.042 (3) Å	θ = 2.4–28.1º
*b* = 9.9782 (8) Å	µ = 0.22 mm^−^^1^
*c* = 25.105 (2) Å	*T* = 296 (2) K
β = 127.917 (1)º	Block, colourless
*V* = 6332.1 (9) Å^3^	0.18 × 0.15 × 0.13 mm
*Z* = 8	

### Data collection

**Table:**

Bruker SMART CCD diffractometer	6212 independent reflections
Radiation source: fine-focus sealed tube	4574 reflections with *I* > 2σ(*I*)
Monochromator: graphite	*R*_int_ = 0.022
*T* = 296(2) K	θ_max_ = 26.0º
ω scans	θ_min_ = 2.1º
Absorption correction: Multi-scan(SADABS; Sheldrick, 2001)	*h* = −25→39
*T*_min_ = 0.962, *T*_max_ = 0.973	*k* = −12→11
17128 measured reflections	*l* = −30→21

### Refinement

**Table:**

Refinement on *F*^2^	Secondary atom site location: difference Fourier map
Least-squares matrix: full	Hydrogen site location: inferred from neighbouring sites
*R*[*F*^2^ > 2σ(*F*^2^)] = 0.069	H-atom parameters constrained
*wR*(*F*^2^) = 0.219	*w* = 1/[σ^2^(*F*_o_^2^) + (0.1169*P*)^2^ + 7.2057*P*] where *P* = (*F*_o_^2^ + 2*F*_c_^2^)/3
*S* = 1.10	(Δ/σ)_max_ < 0.001
6212 reflections	Δρ_max_ = 0.80 e Å^−^^3^
388 parameters	Δρ_min_ = −0.57 e Å^−^^3^
44 restraints	Extinction correction: none
Primary atom site location: structure-invariant direct methods	

### Special details

**Table:**

Geometry. All e.s.d.'s (except the e.s.d. in the dihedral angle between two l.s. planes) are estimated using the full covariance matrix. The cell e.s.d.'s are taken into account individually in the estimation of e.s.d.'s in distances, angles and torsion angles; correlations between e.s.d.'s in cell parameters are only used when they are defined by crystal symmetry. An approximate (isotropic) treatment of cell e.s.d.'s is used for estimating e.s.d.'s involving l.s. planes.
Refinement. Refinement of *F*^2^ against ALL reflections. The weighted *R*-factor *wR* and goodness of fit *S* are based on *F*^2^, conventional *R*-factors *R* are based on *F*, with *F* set to zero for negative *F*^2^. The threshold expression of *F*^2^ > σ(*F*^2^) is used only for calculating *R*-factors(gt) *etc*. and is not relevant to the choice of reflections for refinement. *R*-factors based on *F*^2^ are statistically about twice as large as those based on *F*, and *R*- factors based on ALL data will be even larger.

### Fractional atomic coordinates and isotropic or equivalent isotropic displacement parameters (Å^2^)

**Table:**

	*x*	*y*	*z*	*U*_iso_*/*U*_eq_	
S1	0.15326 (3)	0.47012 (8)	0.46771 (4)	0.0531 (3)	
S2	0.06920 (4)	0.72487 (9)	0.64731 (6)	0.0655 (3)	
C1	0.14805 (12)	0.2922 (3)	0.45787 (15)	0.0464 (7)	
C2	0.10216 (14)	0.2310 (3)	0.40203 (16)	0.0539 (8)	
C3	0.10074 (16)	0.0918 (4)	0.39911 (18)	0.0635 (9)	
H3A	0.0703	0.0502	0.3626	0.076*	
C4	0.1424 (2)	0.0131 (4)	0.4479 (2)	0.0757 (9)	
C5	0.18654 (16)	0.0764 (4)	0.50242 (19)	0.0637 (9)	
H5A	0.2148	0.0242	0.5361	0.076*	
C6	0.19082 (13)	0.2140 (3)	0.50937 (17)	0.0546 (8)	
C7	0.24128 (15)	0.2685 (4)	0.57208 (19)	0.0734 (10)	
H7A	0.2644	0.1956	0.5992	0.110*	
H7B	0.2334	0.3194	0.5974	0.110*	
H7C	0.2583	0.3253	0.5597	0.110*	
C8	0.1398 (2)	−0.1383 (4)	0.4422 (2)	0.0849 (10)	
H8A	0.1062	−0.1645	0.4014	0.127*	
H8B	0.1439	−0.1763	0.4803	0.127*	
H8C	0.1676	−0.1700	0.4413	0.127*	
C9	0.05315 (15)	0.3038 (5)	0.34304 (18)	0.0761 (11)	
H9A	0.0273	0.2393	0.3114	0.114*	
H9B	0.0627	0.3598	0.3210	0.114*	
H9C	0.0385	0.3580	0.3595	0.114*	
C10	0.10008 (13)	0.4624 (3)	0.51695 (17)	0.0581 (8)	
H10A	0.0998	0.3656	0.5203	0.070*	
H10B	0.0691	0.4888	0.4724	0.070*	
C11	0.09849 (17)	0.5273 (4)	0.5705 (2)	0.0732 (10)	
H11A	0.0740	0.4792	0.5740	0.088*	
H11B	0.1333	0.5236	0.6140	0.088*	
C12	0.08133 (16)	0.6696 (4)	0.55154 (18)	0.0698 (10)	
H12A	0.0432	0.6719	0.5171	0.084*	
H12B	0.0972	0.7070	0.5321	0.084*	
C13	0.05511 (12)	0.8878 (3)	0.66060 (17)	0.0512 (7)	
C14	0.08566 (13)	0.9494 (4)	0.72467 (17)	0.0554 (8)	
C15	0.07141 (15)	1.0790 (4)	0.7284 (2)	0.0718 (10)	
H15A	0.0910	1.1211	0.7702	0.086*	
C16	0.03042 (19)	1.1470 (5)	0.6740 (3)	0.1001 (12)	
C17	0.00179 (16)	1.0841 (4)	0.6122 (2)	0.0822 (12)	
H17A	−0.0259	1.1306	0.5745	0.099*	
C18	0.01232 (15)	0.9547 (5)	0.6035 (2)	0.0743 (9)	
C19	−0.02361 (16)	0.8980 (5)	0.5329 (2)	0.0848 (10)	
H19A	−0.0494	0.9641	0.5028	0.127*	
H19B	−0.0413	0.8201	0.5326	0.127*	
H19C	−0.0030	0.8737	0.5185	0.127*	
C20	0.0171 (2)	1.2887 (5)	0.6807 (3)	0.1087 (13)	
H20A	0.0404	1.3153	0.7273	0.163*	
H20B	−0.0188	1.2922	0.6646	0.163*	
H20C	0.0215	1.3484	0.6545	0.163*	
C21	0.13243 (15)	0.8889 (5)	0.78906 (18)	0.0743 (11)	
H21A	0.1462	0.9517	0.8253	0.111*	
H21B	0.1593	0.8679	0.7844	0.111*	
H21C	0.1217	0.8085	0.7987	0.111*	
C22	0.14972 (14)	0.8142 (4)	0.65501 (19)	0.0644 (9)	
H22A	0.1754	0.7424	0.6717	0.077*	
H22B	0.1547	0.8552	0.6936	0.077*	
C23	0.16017 (16)	0.9152 (4)	0.62171 (19)	0.0692 (10)	
H23A	0.1295	0.9732	0.5946	0.083*	
H23B	0.1647	0.8699	0.5914	0.083*	
C24	0.20845 (15)	1.0008 (4)	0.67022 (18)	0.0644 (9)	
H24A	0.2389	0.9430	0.6991	0.077*	
H24B	0.2152	1.0570	0.6447	0.077*	
C25	0.16924 (18)	1.1983 (4)	0.68724 (19)	0.0795 (9)	
C26	0.17520 (14)	1.2568 (3)	0.74571 (17)	0.0596 (8)	
C27	0.15189 (16)	1.3666 (4)	0.7500 (2)	0.0748 (11)	
H27A	0.1280	1.4190	0.7123	0.090*	
C28	0.16498 (18)	1.3968 (4)	0.8121 (2)	0.0789 (11)	
H28A	0.1497	1.4712	0.8162	0.095*	
C29	0.20005 (17)	1.3201 (4)	0.8680 (2)	0.0719 (10)	
H29A	0.2080	1.3429	0.9093	0.086*	
C30	0.22369 (14)	1.2090 (4)	0.86355 (18)	0.0636 (9)	
H30A	0.2474	1.1565	0.9013	0.076*	
C31	0.21105 (12)	1.1789 (3)	0.80191 (16)	0.0528 (7)	
C32	0.22889 (12)	1.0682 (3)	0.78088 (16)	0.0522 (7)	
O1	0.20542 (11)	0.5116 (3)	0.49361 (14)	0.0727 (7)	
O2	0.11048 (11)	0.5309 (3)	0.40790 (13)	0.0741 (7)	
O3	0.10576 (13)	0.6647 (3)	0.71039 (18)	0.0900 (9)	
O4	0.14220 (13)	1.2330 (3)	0.62932 (13)	0.0890 (8)	
O5	0.26026 (10)	0.9802 (3)	0.81456 (12)	0.0691 (7)	
O6	0.02164 (12)	0.6529 (3)	0.59969 (18)	0.0939 (10)	
N1	0.20210 (11)	1.0851 (3)	0.71198 (13)	0.0566 (7)	
N2	0.09614 (12)	0.7566 (3)	0.61035 (16)	0.0662 (8)	
N3	0.14813 (10)	0.5046 (3)	0.52664 (13)	0.0523 (6)	
H3B	0.1729	0.5466	0.5626	0.063*	

### Atomic displacement parameters (Å^2^)

**Table:**

	*U*^11^	*U*^22^	*U*^33^	*U*^12^	*U*^13^	*U*^23^
S1	0.0687 (5)	0.0447 (4)	0.0570 (5)	0.0021 (4)	0.0443 (4)	0.0024 (3)
S2	0.0762 (6)	0.0477 (5)	0.0974 (7)	−0.0042 (4)	0.0659 (6)	−0.0118 (5)
C1	0.0573 (17)	0.0451 (15)	0.0488 (16)	0.0052 (13)	0.0387 (15)	0.0006 (13)
C2	0.0663 (19)	0.0585 (19)	0.0489 (16)	0.0028 (15)	0.0414 (16)	−0.0046 (14)
C3	0.082 (2)	0.061 (2)	0.0574 (19)	−0.0106 (18)	0.0483 (19)	−0.0156 (16)
C4	0.127 (3)	0.0491 (15)	0.094 (2)	0.0034 (17)	0.089 (2)	−0.0075 (15)
C5	0.085 (2)	0.0552 (19)	0.068 (2)	0.0204 (18)	0.056 (2)	0.0112 (17)
C6	0.0643 (19)	0.0556 (18)	0.0589 (18)	0.0071 (15)	0.0454 (17)	0.0024 (15)
C7	0.060 (2)	0.078 (3)	0.062 (2)	0.0080 (19)	0.0281 (18)	0.0070 (19)
C8	0.131 (3)	0.0509 (15)	0.098 (2)	0.0032 (17)	0.083 (2)	−0.0078 (15)
C9	0.064 (2)	0.090 (3)	0.054 (2)	0.005 (2)	0.0263 (18)	−0.0025 (19)
C10	0.0620 (19)	0.0545 (18)	0.0641 (19)	−0.0097 (15)	0.0420 (17)	−0.0172 (15)
C11	0.079 (2)	0.067 (2)	0.087 (3)	−0.0039 (19)	0.058 (2)	−0.010 (2)
C12	0.069 (2)	0.076 (2)	0.065 (2)	0.0016 (19)	0.0412 (19)	−0.0101 (16)
C13	0.0510 (16)	0.0481 (16)	0.0657 (19)	0.0003 (13)	0.0415 (16)	−0.0056 (14)
C14	0.0531 (17)	0.062 (2)	0.0628 (19)	0.0002 (15)	0.0417 (16)	−0.0057 (16)
C15	0.067 (2)	0.069 (2)	0.089 (3)	−0.0076 (18)	0.053 (2)	−0.029 (2)
C16	0.083 (2)	0.068 (2)	0.151 (3)	0.0135 (17)	0.073 (2)	−0.015 (2)
C17	0.061 (2)	0.074 (2)	0.098 (3)	0.0204 (19)	0.042 (2)	0.009 (2)
C18	0.0549 (15)	0.093 (2)	0.0681 (17)	0.0012 (15)	0.0343 (14)	−0.0060 (16)
C19	0.0613 (16)	0.097 (2)	0.0718 (17)	0.0007 (15)	0.0288 (14)	−0.0062 (16)
C20	0.088 (2)	0.070 (2)	0.155 (3)	0.0145 (17)	0.069 (2)	−0.015 (2)
C21	0.070 (2)	0.099 (3)	0.059 (2)	0.007 (2)	0.0428 (19)	0.004 (2)
C22	0.065 (2)	0.062 (2)	0.077 (2)	0.0052 (17)	0.0488 (19)	−0.0013 (18)
C23	0.079 (2)	0.071 (2)	0.069 (2)	−0.0127 (19)	0.051 (2)	−0.0134 (18)
C24	0.068 (2)	0.071 (2)	0.067 (2)	−0.0084 (18)	0.0480 (19)	−0.0093 (18)
C25	0.1013 (19)	0.0670 (16)	0.0558 (13)	0.0131 (14)	0.0410 (14)	0.0110 (12)
C26	0.0613 (19)	0.0478 (17)	0.0597 (19)	−0.0017 (15)	0.0321 (17)	0.0019 (14)
C27	0.077 (2)	0.054 (2)	0.082 (3)	0.0068 (18)	0.043 (2)	0.0018 (18)
C28	0.094 (3)	0.051 (2)	0.108 (3)	0.002 (2)	0.070 (3)	−0.009 (2)
C29	0.087 (3)	0.063 (2)	0.081 (3)	−0.018 (2)	0.059 (2)	−0.020 (2)
C30	0.064 (2)	0.065 (2)	0.0557 (19)	−0.0042 (17)	0.0340 (17)	−0.0022 (16)
C31	0.0471 (16)	0.0491 (17)	0.0546 (17)	−0.0055 (13)	0.0274 (14)	−0.0026 (14)
C32	0.0422 (15)	0.0573 (18)	0.0510 (16)	−0.0027 (14)	0.0255 (14)	0.0013 (14)
O1	0.0816 (17)	0.0666 (16)	0.0918 (18)	−0.0112 (13)	0.0644 (15)	−0.0050 (14)
O2	0.0906 (18)	0.0586 (15)	0.0659 (15)	0.0090 (13)	0.0444 (14)	0.0070 (12)
O3	0.115 (2)	0.0606 (16)	0.125 (3)	0.0143 (16)	0.090 (2)	0.0101 (17)
O4	0.1081 (18)	0.0715 (15)	0.0585 (12)	0.0151 (13)	0.0364 (13)	0.0111 (11)
O5	0.0614 (14)	0.0711 (16)	0.0638 (14)	0.0187 (12)	0.0329 (12)	0.0094 (12)
O6	0.096 (2)	0.0750 (18)	0.146 (3)	−0.0360 (16)	0.092 (2)	−0.0488 (19)
N1	0.0579 (15)	0.0545 (15)	0.0524 (15)	−0.0013 (12)	0.0314 (13)	−0.0031 (12)
N2	0.0697 (18)	0.0615 (17)	0.088 (2)	−0.0128 (14)	0.0592 (17)	−0.0233 (14)
N3	0.0554 (15)	0.0481 (14)	0.0540 (14)	−0.0052 (11)	0.0339 (13)	−0.0116 (11)

### Geometric parameters (Å, °)

**Table:**

S1—O2	1.404 (3)	C15—H15A	0.9300
S1—O1	1.432 (3)	C16—C17	1.375 (7)
S1—N3	1.623 (3)	C16—C20	1.516 (6)
S1—C1	1.786 (3)	C17—C18	1.385 (6)
S2—O3	1.400 (3)	C17—H17A	0.9300
S2—O6	1.424 (3)	C18—C19	1.510 (5)
S2—N2	1.640 (3)	C19—H19A	0.9600
S2—C13	1.773 (3)	C19—H19B	0.9600
C1—C2	1.403 (5)	C19—H19C	0.9600
C1—C6	1.408 (4)	C20—H20A	0.9600
C2—C3	1.390 (5)	C20—H20B	0.9600
C2—C9	1.526 (5)	C20—H20C	0.9600
C3—C4	1.374 (6)	C21—H21A	0.9600
C3—H3A	0.9300	C21—H21B	0.9600
C4—C5	1.377 (6)	C21—H21C	0.9600
C4—C8	1.515 (5)	C22—N2	1.473 (5)
C5—C6	1.379 (5)	C22—C23	1.474 (5)
C5—H5A	0.9300	C22—H22A	0.9700
C6—C7	1.503 (5)	C22—H22B	0.9700
C7—H7A	0.9600	C23—C24	1.513 (5)
C7—H7B	0.9600	C23—H23A	0.9700
C7—H7C	0.9600	C23—H23B	0.9700
C8—H8A	0.9600	C24—N1	1.453 (4)
C8—H8B	0.9600	C24—H24A	0.9700
C8—H8C	0.9600	C24—H24B	0.9700
C9—H9A	0.9600	C25—O4	1.199 (4)
C9—H9B	0.9600	C25—N1	1.402 (5)
C9—H9C	0.9600	C25—C26	1.479 (6)
C10—N3	1.465 (4)	C26—C27	1.367 (5)
C10—C11	1.520 (5)	C26—C31	1.386 (5)
C10—H10A	0.9700	C27—C28	1.375 (6)
C10—H10B	0.9700	C27—H27A	0.9300
C11—C12	1.491 (6)	C28—C29	1.371 (6)
C11—H11A	0.9700	C28—H28A	0.9300
C11—H11B	0.9700	C29—C30	1.385 (5)
C12—N2	1.516 (4)	C29—H29A	0.9300
C12—H12A	0.9700	C30—C31	1.371 (5)
C12—H12B	0.9700	C30—H30A	0.9300
C13—C18	1.402 (5)	C31—C32	1.482 (5)
C13—C14	1.410 (5)	C32—O5	1.205 (4)
C14—C15	1.393 (5)	C32—N1	1.390 (4)
C14—C21	1.498 (5)	N3—H3B	0.8600
C15—C16	1.359 (7)		
			
O2—S1—O1	117.42 (17)	C17—C16—C20	120.9 (5)
O2—S1—N3	107.70 (16)	C16—C17—C18	123.0 (4)
O1—S1—N3	105.58 (15)	C16—C17—H17A	118.5
O2—S1—C1	109.56 (15)	C18—C17—H17A	118.5
O1—S1—C1	109.49 (15)	C17—C18—C13	117.6 (4)
N3—S1—C1	106.49 (14)	C17—C18—C19	116.8 (4)
O3—S2—O6	116.9 (2)	C13—C18—C19	125.6 (4)
O3—S2—N2	110.94 (18)	C18—C19—H19A	109.5
O6—S2—N2	106.63 (18)	C18—C19—H19B	109.5
O3—S2—C13	108.23 (17)	H19A—C19—H19B	109.5
O6—S2—C13	110.82 (17)	C18—C19—H19C	109.5
N2—S2—C13	102.29 (16)	H19A—C19—H19C	109.5
C2—C1—C6	120.5 (3)	H19B—C19—H19C	109.5
C2—C1—S1	121.5 (2)	C16—C20—H20A	109.5
C6—C1—S1	118.0 (2)	C16—C20—H20B	109.5
C3—C2—C1	117.9 (3)	H20A—C20—H20B	109.5
C3—C2—C9	116.3 (3)	C16—C20—H20C	109.5
C1—C2—C9	125.8 (3)	H20A—C20—H20C	109.5
C4—C3—C2	122.8 (4)	H20B—C20—H20C	109.5
C4—C3—H3A	118.6	C14—C21—H21A	109.5
C2—C3—H3A	118.6	C14—C21—H21B	109.5
C3—C4—C5	117.7 (3)	H21A—C21—H21B	109.5
C3—C4—C8	121.3 (4)	C14—C21—H21C	109.5
C5—C4—C8	121.0 (4)	H21A—C21—H21C	109.5
C4—C5—C6	123.0 (3)	H21B—C21—H21C	109.5
C4—C5—H5A	118.5	N2—C22—C23	113.6 (3)
C6—C5—H5A	118.5	N2—C22—H22A	108.9
C5—C6—C1	118.1 (3)	C23—C22—H22A	108.9
C5—C6—C7	116.9 (3)	N2—C22—H22B	108.9
C1—C6—C7	125.1 (3)	C23—C22—H22B	108.9
C6—C7—H7A	109.5	H22A—C22—H22B	107.7
C6—C7—H7B	109.5	C22—C23—C24	113.9 (3)
H7A—C7—H7B	109.5	C22—C23—H23A	108.8
C6—C7—H7C	109.5	C24—C23—H23A	108.8
H7A—C7—H7C	109.5	C22—C23—H23B	108.8
H7B—C7—H7C	109.5	C24—C23—H23B	108.8
C4—C8—H8A	109.5	H23A—C23—H23B	107.7
C4—C8—H8B	109.5	N1—C24—C23	112.4 (3)
H8A—C8—H8B	109.5	N1—C24—H24A	109.1
C4—C8—H8C	109.5	C23—C24—H24A	109.1
H8A—C8—H8C	109.5	N1—C24—H24B	109.1
H8B—C8—H8C	109.5	C23—C24—H24B	109.1
C2—C9—H9A	109.5	H24A—C24—H24B	107.9
C2—C9—H9B	109.5	O4—C25—N1	123.8 (4)
H9A—C9—H9B	109.5	O4—C25—C26	130.2 (4)
C2—C9—H9C	109.5	N1—C25—C26	105.9 (3)
H9A—C9—H9C	109.5	C27—C26—C31	121.3 (4)
H9B—C9—H9C	109.5	C27—C26—C25	130.6 (3)
N3—C10—C11	109.5 (3)	C31—C26—C25	108.0 (3)
N3—C10—H10A	109.8	C26—C27—C28	117.7 (4)
C11—C10—H10A	109.8	C26—C27—H27A	121.2
N3—C10—H10B	109.8	C28—C27—H27A	121.2
C11—C10—H10B	109.8	C29—C28—C27	121.6 (4)
H10A—C10—H10B	108.2	C29—C28—H28A	119.2
C12—C11—C10	109.3 (3)	C27—C28—H28A	119.2
C12—C11—H11A	109.8	C28—C29—C30	120.6 (4)
C10—C11—H11A	109.8	C28—C29—H29A	119.7
C12—C11—H11B	109.8	C30—C29—H29A	119.7
C10—C11—H11B	109.8	C31—C30—C29	118.0 (3)
H11A—C11—H11B	108.3	C31—C30—H30A	121.0
C11—C12—N2	113.7 (3)	C29—C30—H30A	121.0
C11—C12—H12A	108.8	C30—C31—C26	120.8 (3)
N2—C12—H12A	108.8	C30—C31—C32	130.9 (3)
C11—C12—H12B	108.8	C26—C31—C32	108.4 (3)
N2—C12—H12B	108.8	O5—C32—N1	124.9 (3)
H12A—C12—H12B	107.7	O5—C32—C31	129.1 (3)
C18—C13—C14	121.0 (3)	N1—C32—C31	105.9 (3)
C18—C13—S2	116.5 (3)	C32—N1—C25	111.7 (3)
C14—C13—S2	122.4 (3)	C32—N1—C24	125.1 (3)
C15—C14—C13	117.0 (3)	C25—N1—C24	123.2 (3)
C15—C14—C21	116.9 (3)	C22—N2—C12	119.1 (3)
C13—C14—C21	126.0 (3)	C22—N2—S2	114.7 (2)
C16—C15—C14	123.4 (4)	C12—N2—S2	118.8 (2)
C16—C15—H15A	118.3	C10—N3—S1	118.2 (2)
C14—C15—H15A	118.3	C10—N3—H3B	120.9
C15—C16—C17	117.9 (4)	S1—N3—H3B	120.9
C15—C16—C20	121.2 (5)		

### Hydrogen-bond geometry (Å, °)

**Table:**

*D*—H···*A*	*D*—H	H···*A*	*D*···*A*	*D*—H···*A*
N3—H3B···O5^i^	0.86	2.53	3.192 (4)	134

Symmetry codes: (i) −*x*+1/2, *y*−1/2, −*z*+3/2.
